# Cross-cultural adaptation and validation in spanish of the malocclusion impact questionnaire (MIQ)

**DOI:** 10.1186/s12955-020-01385-1

**Published:** 2020-05-19

**Authors:** Bárbara Hope, Carlos Zaror, Paulo Sandoval, Mario Garay, David L. Streiner

**Affiliations:** 1 Master’s Program in Dentistry, Faculty of Dentistry, Universidad de La Frontera, Temuco, Chile; 2 Universidad San Sebastián, Facultad de Odontología, Puerto Montt, Chile; 3 Department of Pediatric Dentistry and Orthodontics, Faculty of Dentistry, Universidad de La Frontera, Manuel Montt #112, Temuco, Chile; 4 Center for Research in Epidemiology, Economics and Oral Public Health (CIEESPO), Faculty of Dentistry, Universidad de La Frontera, Temuco, Chile; 5 Department of Psychiatry and Behavioral Neurosciences, McMaster University, Hamilton, ON Canada

**Keywords:** Malocclusion, Quality of life, Psychometrics, Outcome assessment, Child

## Abstract

**Background:**

The Malocclusion Impact Questionnaire (MIQ) is a condition-specific measure that assesses the impact of malocclusion on Oral Health-Related Quality of Life (OHRQoL). The aim of this study was to cross-culturally adapt the original version of MIQ into Spanish and to assess the acceptability, reliability and validity of this version in the Chilean population.

**Methods:**

The MIQ was cross-culturally adapted for the Spanish language for Chile using recommended standards for the linguistic validation of instruments. To assess its psychometric properties, a cross-sectional study was carried out with 219 children aged 10 to 16 years from public schools in Puerto Montt, Chile, who completed the Chilean versions of the MIQ (MIQ_Ch_) and the Child Perceptions Questionnaire 11–14 (CPQ_11–14_). The presence and severity of malocclusions was determined through the Dental Aesthetic Index by a trained dentist. The MIQ was administrated a second time two weeks later. The reliability of the scale was assessed by analysis of its internal consistency (Cronbach’s alpha) and reproducibility (Intraclass correlation coefficient – ICC). The validity of the construct was assessed by confirmatory factor analysis and known groups method. Criterion validity was assessed by calculating the Spearman correlation with the CPQ_11–14_.

**Results:**

The content comparison of the back-translation with the original MIQ showed that all items except two were conceptually and linguistically equivalent. The cognitive debriefing showed a suitable understanding of the Chilean version. The MIQ_Ch_ demonstrated good reliability, with Cronbach’s alpha coefficient of 0.85 and ICC of 0.91. A moderate correlation was found between the MIQ_Ch_ and CPQ_11–14_ (0.58). In the known groups comparison, children who felt that their teeth bothered them and/or affected their life obtained significantly higher scores on the MIQ_Ch_. The OHRQoL was worse when the severity of the malocclusion was greater (*p* = 0.03).

**Conclusions:**

The results support the applicability, reliability and validity of the Spanish version of MIQ for assessing OHRQoL in Chilean children with malocclusions.

## Background

Malocclusions are a highly prevalent oral disease, ranked in third place worldwide by the WHO, following dental caries and periodontal disease [1]. In Chile, there are several studies that support this finding, with prevalence rates ranging from 38.3 to 96.2% in children and adolescents [2–5]. It is well documented that malocclusions can lead to severe consequences in those who suffer from them. These consequences can be functional, aesthetic, emotional and social [6, 7]. Among the daily life limitations that children and adolescents have to face because of their malocclusions are: altering the way they smile or laugh, social isolation, avoiding taking their photographs, being picked on or bullied, having a lack of confidence that translates in difficulties in making friends or fitting in with their peers, having difficulties when biting certain types of food, and being more prone to suffer from alveolar dental trauma [8–10].

Because of these consequences, malocclusions have an impact on the Oral Health Related Quality of Life (OHRQoL), a “multidimensional construct that includes a subjective evaluation of the individual’s oral health, functional well-being, emotional well-being, expectations and satisfaction with care, and sense of self.” [11]. This concept is related to the impact that oral health or disease has on the patient’s daily activities, wellbeing and quality of life [12]. When it comes to malocclusions, studies suggest that the most serious impact in children’s and adolescent’s OHRQoL has to do with the psychosocial aspect, in comparison with oral symptoms and functional limitations [13, 14].

Taking into consideration the psychosocial model, clinical indexes alone are not enough for describing health status, because they do not reveal the impact of oral diseases on the patient’s psychosocial wellbeing. Measuring both functional and psychological outcomes of oral conditions, in addition to clinical indicators, can provide a more complete oral health evaluation [11]. This way, effective oral health programs can be developed, because by starting with the evaluation of the patient-perceived needs, it is possible to determine the level of care needed and the effectiveness of treatment strategies [15]. Success in malocclusion’s orthodontic treatment has historically been measured only from the clinician’s point of view, taking into consideration strict occlusal measurements and parameters, but leaving out the patient’s needs and reasons to seek treatment in the first place [16, 17]. Using OHRQoL as an outcome measuring tool is congruent with patient-centered care, and is crucial to understand treatment effectiveness from the patient’s point of view, as well as the interrelations between oral health specifics and general health over time [18–20].

Because of this, researchers are using specific OHRQoL measure instruments, in order to assess more accurately the impact oral conditions and related interventions have on it. Several children-focused scales have been developed and validated, which has improved the capture of the consequences oral diseases have in this population [21, 22]. The first child and adolescent specific scale to measure OHRQoL and treatment experience was the Child Perceptions Questionnaire (CPQ_11–14_). Even though this scale shows that malocclusions do affect everyday activities and behaviors, it demonstrates only a modest association. In fact, most current OHRQoL scales for children focus mainly on dental caries and its consequences, leaving little or no room for malocclusions impact measures, which shows that the scales available today do not apply to children with orthodontic needs [16].

Recently, the Malocclusion Impact Questionnaire (MIQ) has been developed, which is a self-administered condition-specific measure of OHRQoL designed to measure the impact that malocclusions have on children aged 10 to 16 years [10, 23]. Validating an existing scale is necessary when it is applied to a population with a different language and culture than the original. It is also more cost-effective than developing a whole new scale, with the added advantage that allows for inter-country, and, therefore, inter-culture comparisons [24]. Since the MIQ was developed in the United Kingdom, and has not been translated into other languages, it is only available in English. Due to its high prevalence in Chile, it is very important to have a validated instrument that measures OHRQoL specifically for malocclusions, because it will allow monitoring the impact they have on young people, assessing the impact of orthodontic treatments implemented as public health policies, and serve as a crucial tool for the development of public health research in this matter. The objective of this study is to develop the Chilean version of MIQ in Spanish and validate it for its use in Chilean population.

## Methods

The Ethics Committee of Universidad de La Frontera approved the study’s protocol (Resolution n° 098/2017) to carry out the cross-cultural adaptation to Spanish of the Malocclusion Impact Questionnaire and its psychometric evaluation in Chilean schoolchildren.

### Malocclusion impact questionnaire (MIQ)

The Malocclusion Impact Questionnaire was the first instrument developed to measure OHRQoL in preadolescents and adolescents in regards to malocclusions. It is a self-administered instrument that consists of two global questions: “Overall, how much do your teeth bother you?”, and “Overall, how much do your teeth affect your life?”; and 17 specific questions divided into four sections: *how your teeth make you feel* (seven items), *how your teeth affect you in specific situations* (four items), *if your teeth make you worried or concerned* (four items), and *other ways your teeth might affect you* (two items). Each item has a 3-point severity or intensity response format, scored 0 = Don’t/Doesn’t; 1 = A bit; 2 = Very/A lot for negatively scored questions or 0 = Very/A lot; 1 = A bit; 2 = Don’t/Doesn’t for positively worded questions. The total score ranges between 0 and 34, obtained by a simple sum of the individual item scores. Higher scores indicate worse OHRQoL. The response categories for the global questions are rated on a 5-point adjectival scale from 0 = *Not at all* to 4 = *Very much*.

### Translation and cultural adaptation

The translation and cultural adaptation into Spanish of the MIQ for its use in the Chilean population followed the guidelines proposed by ISPOR’s Translation and Cultural Adaptation Good Practice Principles [25]. Two independent translators conducted forward translation of the MIQ from the original language (English) to the target language (Spanish). The translators were Spanish native speakers fluent in English. They were asked to maintain the conceptual equivalence of the original version, rather than literal equivalence. Afterwards, the reconciliation process was carried out by an expert panel that included both translators, the project manager and other disciplinary experts (two pediatric dentists and two methodological experts). Discrepancies were discussed until a unified first version was obtained.

This first version of the Chilean MIQ (MIQ_Ch_) was translated back into English, separately, by two English native speakers fluent in Spanish. The equivalence between the original version and back-translation was evaluated by the expert panel who rated the items as: A: conceptually and linguistically equivalent to the original item; B: functionally equivalent, but with grammatical differences; or C: equivalence is not obvious. Subsequently, the panel composed of researchers and translators met to discuss the discrepancies in equivalence (categories B and C), and to find an equivalent version in Spanish. The report on equivalence between original and back-translated versions was sent to the author of the original scale for evaluation.

Finally, a cognitive debriefing process using the harmonized instrument was carried out on a group of 13 Chilean schoolchildren between 10 and 16 years-old to evaluate any difficulties in the understanding of the preliminary version.

### Study participants

To determine the psychometric properties of the Chilean version of MIQ, a cross-sectional study was carried out from May 2018 to November 2018 in a public school in the city of Puerto Montt, in the south of Chile. This urban school is funded by the Chilean government and receives children of 4 to 16 years old, mainly of low socioeconomic status. Although there are public dental services for this population, access to orthodontic treatment is limited to the most severe cases. Participants between 10 and 16 years of age with a self-report of “needing braces” were included. Participants who underwent previous orthodontic treatment or who used orthodontic devices were excluded. Those who suffered systemic diseases, disabilities or learning problems were also excluded. Both informed consents signed by the children’s parents and informed consent signed by the schoolchildren themselves were obtained.

### Measures

The children/adolescents were examined in order to determine the presence and severity of malocclusions using the Dental Aesthetic Index (DAI) [26]. The DAI consists of 10 occlusal characteristics related to dentofacial anomalies according to the three components of the dentition: spacing, crowding and occlusion. Overjet, negative overjet, tooth loss, diastema, anterior open bite, anterior crowding, anterior diastema, width of the anterior irregularities (mandible and maxilla) and antero-posterior spring relationship are evaluated by clinical examination and the use of a University of North Carolina-15 periodontal probe. The DAI score is obtained by multiplying the value obtained by the weighting factor (regression coefficient) for each of the components and the subsequent sum of the results obtained, to which a constant is added. The final score classifies the patient into: normal occlusion, mild malocclusion, definitive malocclusion, severe malocclusion and very severe malocclusion [26]. Clinical examination was carried out by two previously calibrated examiners (kappa = 0.91). Then, the participants were asked to answer the Chilean version of MIQ, and a previously validated Chilean version of CPQ_11–14_ [27]. CPQ_11–14_ is a 37-question self-administered measure of OHRQoL for children between 11 and 14 years of age that includes four domains: oral symptoms, functional limitations, emotional and social wellbeing. The questions focus on the frequency that certain oral events happened to the child in the past 3 months. The items have 5 rated response options ranging from a score of 0 to 4 on a Likert scale of “never” to “every day or almost every day”. The total score ranges between 0 and 148, with higher scores indicating worse OHRQoL [27]. The examiners were blinded to the questionnaire’s responses. Findings were recorded in a customized clinical record that included age, gender, socioeconomic status and oral health status.

The sample size was calculated following Terwee’s recommendations [28], where 4–10 subjects are needed per item, with an absolute minimum of 100 subjects. Considering the highest number of participants needed, and a 20% assumption of missing answers, the calculated sample size was 204 children/adolescents.

After two to three weeks, all participants were asked to answer again the Chilean version of MIQ, in order to obtain the instrument’s test-retest reliability.

### Statistical analysis

Data analysis included descriptive statistics to examine the distribution of the MIQ_Ch_ scores. Mean, standard deviations, score range, floor and ceiling effects (percentage of patients with minimum and maximum theoretical scores, respectively) were calculated. Small floor or ceiling effects (< 15%) are considered acceptable [28]. Reliability was assessed by tests of internal consistency using Cronbach’s alpha and the test-retest reproducibility through the intraclass correlation coefficient (ICC), in which the MIQ_Ch_ was re-administered to all participants between 2 and 3 weeks after they answered the questionnaire the first time. Values > 0.8 for both measures are considered acceptable for comparison between groups.

Confirmatory factorial analysis (CFA) was carried out to confirm the single dimension of the Chilean version proposed by developers of the MIQ. Comparative Fit Index (CFI), the Tucker-Lewis Index (TLI) and the Root Mean Square Error of Approximation (RMSEA) were calculated to evaluate the model. For the CFI and TLI, values equal or greater than 0.90 suggest an acceptable fit of the model. In RMSEA, values below 0.08 indicates an acceptable model fit, with an ideal value below 0.05 [29].

Criterion validity was assessed by examining the correlation between the total scores of MIQ_Ch_ with the total scores of the CPQ_11–14_ using the Spearman correlation coefficient, interpreted as: negligible relationship when r is < 0.20; weak when 0.20–0.40; moderate when 0.40–0.60; strong-moderate when 0.60–0.80; and strong relationship when > 0.80 [30].

Construct validity was based on known groups by comparing the MIQ_Ch_ scores with the severity of malocclusion and global questions of the MIQ_Ch_. We hypothesized that children with severe malocclusion and those whose overall oral health was worse would have a poorer OHRQoL. The differences in MIQ_Ch_ scores among these groups were assessed using Kruskal-Wallis tests. The data analyses were performed using Stata 15 [Stata Corp, College Station, TX, USA].

## Results

### Transcultural adaptation

For content comparison between back-translation and the original version, the expert panel rated all items as A (conceptually and linguistically equivalent) or B (functionally equivalent, but with grammatical differences), except for two items which were rated as C (equivalence is not obvious). In relation to the first item, “Being bullied” (item 13), in Chile, the term “bullying” does not refer to teasing and making fun of others, but it does imply verbal and physical violence. Thus, after discussion by the group of researchers and translators, the expression was modified to “being made fun of”. For item 17, “Because of the way my teeth meet...” the term “meet” was replaced by “bite”. In spite its redundancy, asking about how their teeth “bite” was widely accepted and understood for the children/adolescents in the cognitive debriefing. The author of the original MIQ reviewed the Spanish and the English back-translated versions without identifying any lack of equivalence regarding the original.

The cognitive interview showed that the instructions, items and response options were easily understood by the children/adolescents. All questionnaires were answered in under 5 mins, and no problems were identified in differentiating between the items or the different response options. No modifications were needed as a result of the cognitive interviews.

### Study of psychometric properties

Four hundred eighteen informed consents were distributed among children between 10 and 16 years old, and 236 were signed by a parent. One child did not give her consent, so she was not included. Out of the remaining 235, after clinical examination, 16 were excluded due to absence of malocclusion or currently being under orthodontic treatment, with a total of 219 subjects included in this study. Table 1 shows the characteristics of the participants. The mean age of the children included was 11.6 years [SD = 1.6], 53% were girls [*n* = 116], and 71.2% had low socio-economic status. Only 6.9% of the children reported that their teeth bothered them and 14.1% that they affected their quality of life quite a bit or very much. Seventy five percent of the participants had a malocclusion that required treatment.

**Table 1:** Demographic and clinical characteristics of the participants

Variables	n [%]
**Age in years [mean ± SD]**	11.6 [1.6]
**Gender**	
Male	103 [47.0]
Female	116 [53.0]
**Socioeconomic status**	
Low	156 [71.2]
Medium-high	63 [28.8]
**Overall, how much do your teeth bother you?**	
Not at all or A little	164 [74.9]
Somewhat	40 [18.2]
Quite a bit or Very much	15 [6.9]
**Overall, how much do your teeth affect your life?**	
Not at all or A little	151 [68.9]
Somewhat	37 [16.9]
Quite a bit or Very much	31 [14.1]
**Malocclusion**	
Mild	55 [25.2]
Definitive-Severe	91 [41.7]
Very severe	72 [33.0]

Table 2 shows distribution statistics and Cronbach’s alpha coefficient of the MIQ_Ch_ and CPQ_11–14_ scales. All items of the MIQ_Ch_ and CPQ_11–14_ were completed by participants. No ceiling effect was observed, but a slight floor effect was observed in the MIQ_Ch_ [4.1%]. The Cronbach’s alpha coefficient was 0.85 for the total score showing a good correlation within items. The Intraclass Correlation Coefficient was 0.91 for the total score indicating good repeatability.

**Table 2:** Descriptive data for the Malocclusion Impact Questionnaire [MIQ] and the Child Perceptions Questionnaire [CPQ11–14]

Scale	Number of items	Observed range	Median [IQR]	Mean [SD]	Percent floor [%]	Percent ceiling [%]	Cronbach’s alpha
**MIQ total score**	17	0–31	7 [8]	8.3 [6.0]	4.1	0.0	0.85
**CPQ11–14 total score**	37	2–96	30	32.8 [17.9]	0.0	0.0	0.89
Oral symptoms	6	0–21	7 [6]	7.1 [3.7]	2.3	0.0	0.62
Functional limitations	9	0–23	7 [8]	8.3 [5.7]	3.2	0.0	0.74
Emotional well-being	9	0–32	8 [9]	8.7 [6.1]	4.6	0.0	0.80
Social well-being	13	0–36	7 [10]	8.7 [7.0]	6.4	0.0	0.78

Floor effect: percentage of patients with minimum score; Ceiling effect: percentage of patients with maximum score

*IQR* Interquartile range, *SD* standard deviation

**Fig. 1:**
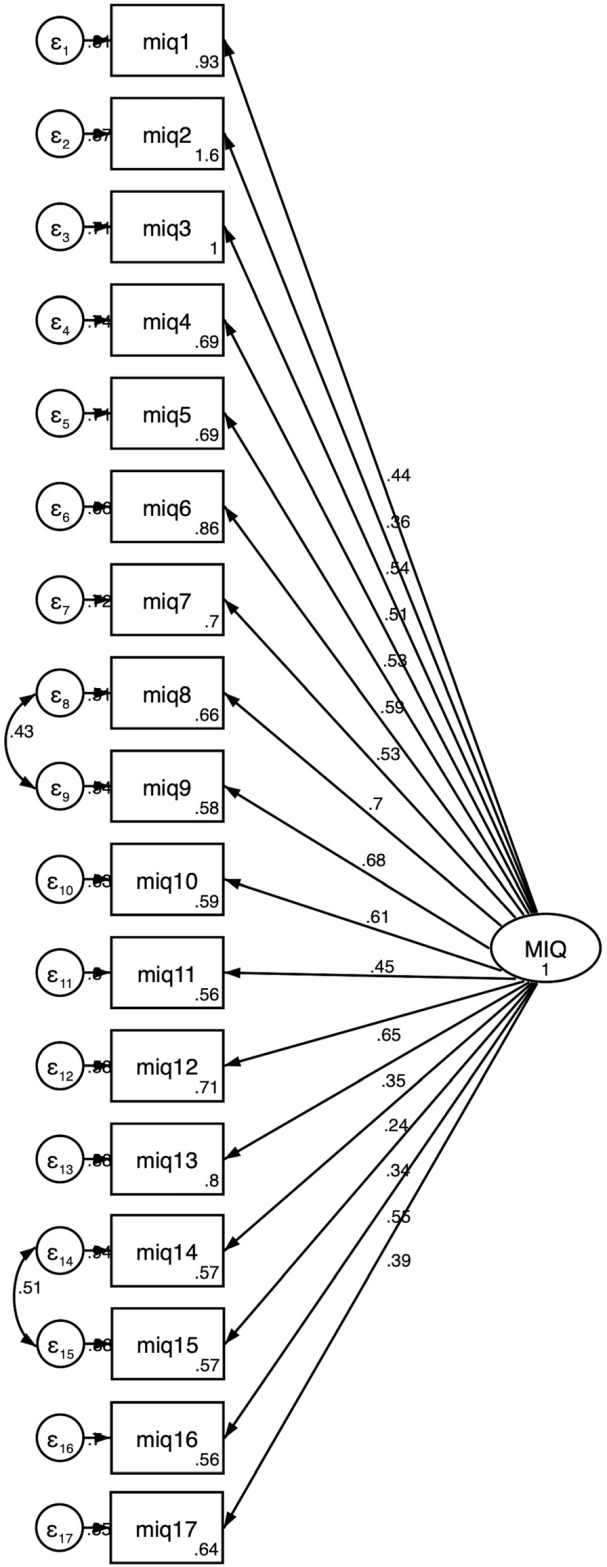
Confirmatory factor analysis on the 17-item of the Malocclusion Impact Questionnaire

Figure 1 shows results of the confirmatory factor analysis in which the 17 items of the MIQ indicate a single latent factor. The values of fit indices were CFI = 0.784, TLI = 0.753 and RMSEA = 0.089. To improve the overall model fit, a Lagrange multiplier test was applied and covariances were incorporated between: item 10 (smiling) and 11 (laughing); and item 16 (making friends) and 17 (fitting in with friends). The new model showed a better goodness of fit indices: CFI = 0.883, TLI = 0.864 and RMSEA = 0.066. However, there were still four items that did not load strongly into the latent factor (factor loading under 0.4): “Good looking”, “Being bullied”, “Making friends”, and “Fitting in with friends”.

Table 3 shows the results for the construct validity based on known groups. Children who felt that their teeth bothered them and or affected their life obtained significantly higher scores on the MIQ_Ch_, meaning a lower quality of life (*p* < 0.01). In addition, the OHRQL was worse when the severity of the malocclusion was greater (*p* = 0.03). The CPQ_11–14_ was not able to discriminate between severity of malocclusion (*p* = 0.751). Finally, the correlation between MIQ_Ch_ and CPQ_11–14_ was moderate (r = 0.58).

**Table 3:** Construct validity of Malocclusion Impact Questionnaire total score based on known groups

Variables	n	Median [IQR]	Mean [SD]	p
**Overall, how much do your teeth bother you?**				
Not at all or A little	164	6 [6]	7.0 [5.0]	< 0.01
Somewhat	40	9 [5.5]	10.3 [5.6]	
Quite a bit or Very much	15	20 [15]	16.9 [8.8]	
**Overall, how much do your teeth affect your life?**				
Not at all or A little	151	6 [7]	6.9 [5.2]	< 0.01
Somewhat	37	9 [6]	10.1 [4.3]	
Quite a bit or Very much	31	12 [18]	12.8 [8.4]	
**Malocclusion**				
Moderate	56	6 [6.5]	6.7 [5.1]	0.03
Definitive-Severe	91	7 [8]	8.5 [6.0]	
Very severe	72	8 [8]	9.4 [6.5]	

*IQR* Interquartile range, *SD* standard deviation

## Discussion

The Chilean version of MIQ showed good psychometric properties, with internal consistency and reproducibility results that ensure the translated instrument’s reliability. It was shown to be valid to discriminate between children with different severities of malocclusion and its results are consistent with those obtained for the original English version.

There was no data loss in the MIQ_Ch_ suggesting that the items making up the scale were well understood and acceptable for the children. Therefore, no interview administration was needed, and no one required assistance to self-complete the questionnaire. This also confirms that the cross-cultural adaptation process was well done and that the Chilean version of the MIQ is conceptually and metrically equivalent, and that the adapted instrument is coherent with the subjects’ self-perception. Self-administration presents advantages, such as lower cost, preservation of participant’s anonymity, and reduction of interviewer bias [31]. Studies with other OHRQoL instruments showed that administration mode (interview versus self-administered) does not influence the instruments’ scores [32, 33]. This is important because, since malocclusions have an impact on the OHRQoL, it is only logical that the patient’s perception is taken into account when deciding the need for orthodontic treatment. Studies show that the malocclusion alone does not determine the impact on OHRQoL, therefore, emotional and social aspects must also be considered [34]. Even parents’ perception about their child’s malocclusion can be misleading. Benson et al. in 2010 showed that, while mother’s and child’s overall opinion on the impact that malocclusions had on the child’s OHRQoL was similar, the parent tended to overestimate the emotional impact, showing that they were more dissatisfied with the appearance of their children’s teeth that the child himself [35], which could lead to unnecessary treatments from the patient’s perspective. This was confirmed by Abreu et al., who also reported a poor agreement between adolescents and their parents/caregivers in rating the impact of malocclusion on adolescents’ OHRQoL [36].

Similar to the original version, no ceiling effect was observed. However, the Chilean version presented slightly higher floor effect (4.1% vs 0.0%) [23]. Despite that, both were less than 15% reflecting a good content coverage.

The reliability of the MIQ_Ch_ assessed by Cronbach’s alpha coefficient and ICC achieved the recommended standard of > 0.70. Benson et al. [23] obtained similar results when initially validating the MIQ, with internal consistency and test retest reproducibility results of 0.90 and 0.78 respectively, compared with this study’s results of 0.85 and 0.91. The New Zealand sample reported higher Cronbach’s alpha coefficient values than ours (0.92) [37].

To the best of our knowledge, there is no previous publication describing the factor structure of the original MIQ; therefore, comparisons with other studies are not possible. Our results of the CFA support the unidimensional structure of the MIQ. While the initial model showed discrepancies between observed values and the values expected under the hypothetical model (original version of the MIQ), it was remedied by adding covariances between errors that reflect all other sources of variance in the items not explained by the construct (Chilean version of MIQ), suggesting the possibility of sub-scales. While the RMSEA value in this sample indicate an acceptable fit, the other fit indices, CFI and TLI, showed slightly less than what is considered acceptable for a good fit of the model (0.883 and 0.864). However, it is important to consider that the indices tests fit in different ways. Although RMSEA is calculated only from the *X*^2^ statistic for the proposed model, the other indices fit by comparing with the null model, which postulates that all manifest variables are uncorrelated, being unlikely in the population. Therefore, to determine whether a given model fits well enough to yield interpretable parameters, RMSEA seems to be a reliable index [38]. In spite of that, four items did not fit well; therefore, the confirmation of this measurement model for the original version of the MIQ would be recommendable.

Two global questions were used to evaluate the construct validity of the measure. These findings were consistent with previous studies in which children who perceived that their teeth bothered or affected their lives had significantly higher mean MIQ scores [23, 37]. Also, the mean score on the MIQ_Ch_ was higher in children who presented a worse severity of the malocclusion, confirming the discriminative ability of the MIQ according to severity of malocclusion.

Conversely, the CPQ_11–14_ was not able to discriminate between severity of malocclusion based on OHRQoL. Marshman et al. reported face and content validity concerns in regards CPQ_11–14_, where some questions were considered not to be relevant or the response format was inadequate to assess OHRQoL in children with malocclusions [39]. A recent meta-analysis showed only very severe malocclusion affected the domains of oral symptoms, emotional well-being and the overall OHRQoL using CPQ [40]. This is relevant since only 33% of our sample had severe malocclusion.

The moderate correlation between MIQ_Ch_ and CPQ_11–14_ suggests that MIQ captures additional information, which is not covered by generic instruments measuring OHRQoL. Generic instruments are unable to measure the impact of the small but important impairment produced for a particular pathology. Diseases may affect different functions and lead to different physical or emotional problems, or affect other aspects of quality of life [41, 42]. Our results are in line with other studies which show that OHRQoL scales are more sensitive than generic scales in measuring the impact of oral problems in children [42, 43]. While specific measures require more time and are more expensive to develop and administer, they usually have a shorter administration burden when compared to generic measures [42]. Nonetheless, when validating the original instrument [23], the authors found a good correlation of 0.75 as opposed to the 0.56 obtained in our study. Therefore, although CPQ is a suitable tool to measure OHRQoL in children, the overall score may not be as sensitive in measuring the impact malocclusions alone have in the OHRQoL. Agou et al. showed that children with low self-esteem and malocclusions had higher CPQ_11–14_ scores than those with malocclusions but with better self-esteem. Therefore, they conclude that self-esteem is a more salient determinant of OHRQoL in children seeking orthodontic treatment. It shows that the impact on OHRQoL that malocclusions alone have cannot be accurately determined with CPQ_11–14_, because there are other factors that influence the child’s perception [34].

The strengths of this study include the sample size, which is greater than the one used in previously conducted validation studies of the MIQ, and because it is the first study performed in a community context.

The main limitation of this study was the homogeneity of the sample studied, with high indices of low socio-economic status, an important determinant of OHRQoL. Since the samples may not fully represent all Chilean children, the association may be distorted. However, this is a priority population for the implementation of public policies, and evaluation of their OHRQoL can provide information to support the decision-making process. Furthermore, the sensitivity to change was not assessed, so further studies are needed to assess the ability of the Chilean version of the MIQ to detect changes over time.

## Conclusions

The results of the present study support the applicability, reliability and validity of the Chilean version of the MIQ in Spanish for assessing OHRQoL in children with malocclusion. Comparison with the study of the psychometric properties of the original instrument showed similar results for validity and reliability, supporting the equivalence with the Spanish for Chile cross-cultural adaptation.

## Data Availability

The datasets used and/or analyzed during the current study are available from the corresponding author upon reasonable request.

## Abbreviations

CFA: Confirmatory factor analysis (EFA)
CPQ_11–14_: Children Perceptions Questionnaire 11–14
DAI: Dental Aesthetic Index EFA Exploratory factor analysis
ICC: Intraclass correlation coefficient
MIQ_ch_: Chilean Malocclusion Impact Questionnaire
OHRQoL: Oral Health-Related Quality of Life
